# Influence of geographical latitude on vitamin D status: cross-sectional results from the BiomarCaRE consortium

**DOI:** 10.1017/S0007114521005080

**Published:** 2022-12-14

**Authors:** Viktor Oskarsson, Mats Eliasson, Veikko Salomaa, Jaakko Reinikainen, Satu Männistö, Luigi Palmieri, Chiara Donfrancesco, Susana Sans, Simona Costanzo, Giovanni de Gaetano, Licia Iacoviello, Giovanni Veronesi, Marco M. Ferrario, Teresa Padro, Barbara Thorand, Cornelia Huth, Tanja Zeller, Stefan Blankenberg, Annie S. Anderson, Hugh Tunstall-Pedoe, Kari Kuulasmaa, Stefan Söderberg

**Affiliations:** 1 Department of Public Health and Clinical Medicine, Umeå University, Umeå 90187, Sweden; 2 Department of Public Health Solutions, Finnish Institute for Health and Welfare, Helsinki, Finland; 3 Department of Cardiovascular, Endocrine-Metabolic Diseases and Aging, Istituto Superiore di Sanità-ISS, Rome, Italy; 4 Catalan Department of Health, Barcelona, Spain; 5 Department of Epidemiology and Prevention, IRCCS Neuromed, Pozzilli, Italy; 6 Department of Medicine and Surgery, University of Insubria, Varese, Italy; 7 Cardiovascular-Program ICCC, Research Institute Hospital Santa Creu i Sant Pau, IIB-Sant Pau, Barcelona, Spain; 8 Institute of Epidemiology, Helmholtz Zentrum München, German Research Center for Environmental Health, Neuherberg, Germany; 9 University Heart and Vascular Center Hamburg, Medical University Hamburg-Eppendorf, Hamburg, Germany; 10 German Center for Cardiovascular Research, Partner Site Hamburg/Lübeck/Kiel, Hamburg, Germany; 11 Centre for Public Health Nutrition Research, University of Dundee, Dundee, Scotland; 12 Institute of Cardiovascular Research, University of Dundee, Dundee, Scotland

**Keywords:** Vitamin D, 25-hydroxyvitamin D, Latitude, Epidemiology, Europe, Population based

## Abstract

Even though sunlight is viewed as the most important determinant of 25-hydroxyvitamin D (25(OH)D) status, several European studies have observed higher 25(OH)D concentrations among north-Europeans than south-Europeans. We studied the association between geographical latitude (derived from ecological data) and 25(OH)D status in six European countries using harmonised immunoassay data from 81 084 participants in the Biomarkers for Cardiovascular Risk Assessment in Europe (BiomarCaRE) project (male sex 48·9 %; median age 50·8 years; examination period 1984–2014). Quantile regression models, adjusted for age, sex, decade and calendar week of sampling and time from sampling to analysis, were used for between-country comparisons. Up until the median percentile, the ordering of countries by 25(OH)D status (from highest to lowest) was as follows: Sweden (at 65·6–63·8°N), Germany (at 48·4°N), Finland (at 65·0–60·2°N), Italy (at 45·6–41·5°N), Scotland (at 58·2–55·1°N) and Spain (at 41·5°N). From the 75th percentile and upwards, Finland had higher values than Germany. As an example, using the Swedish cohort as a comparator, the median 25(OH)D concentration was 3·03, 3·28, 5·41, 6·54 and 9·28 ng/ml lower in the German, Finnish, Italian, Scottish and Spanish cohort, respectively (*P*-value < 0·001 for all comparisons). The ordering of countries was highly consistent in subgroup analyses by sex, age, and decade and season of sampling. In conclusion, we confirmed the previous observation of a north-to-south gradient of 25(OH)D status in Europe, with higher percentile values among north-Europeans than south-Europeans.

Vitamin D is a nutrient that has spurred substantial scientific debate over the last 30 years, mostly due to its role in musculoskeletal health^(1)^. In addition, multiple studies have reported on an association between vitamin D status and risk of cancer, cardiac disease, stroke and diabetes (as reviewed by Mondul *et al*.^(2)^, Rai *et al*.^(3)^, Zhou *et al*.^(4)^ and Lips *et al.* ^(5)^, respectively) as well as with overall mortality (as reviewed by Heath *et al*.^(6)^). It is, however, unclear whether these are causal associations, given that randomised clinical trials and other controlled studies have yielded mostly null results^(7–10)^. Recently, it was also hypothesised that vitamin D status might affect the severity of COVID-19^(11)^.

Vitamin D status is defined by the total serum or plasma 25-hydroxyvitamin D (25(OH)D) concentration, of which the two major forms – with equal biological importance – are D_2_ (ingested via plant-based or fortified foods) and D_3_ (synthesised in human skin and/or ingested via animal-based or fortified foods)^(12)^. The most important determinant of 25(OH)D status is thought to be sunlight, which initiates the cutaneous synthesis of 25(OH)D^(13)^. North-Europeans have, therefore, often been viewed as more at risk for vitamin D insufficiency than other Europeans, mainly because the cutaneous synthesis of 25(OH)D is virtually undetectable from October to March at geographical latitudes above 50°N^(14)^. However, in several European multicentre studies (*n* 824 to 55 844 participants) with harmonised (i.e. analysed in the same lab and with the same assay method) or standardised (i.e. using a Vitamin D Standardisation Program protocol^(15)^) data on 25(OH)D, there has been a positive association between geographical latitude and 25(OH)D status^(16–23)^; that is, the opposite of what is naively expected if sunlight is the main determinant of 25(OH)D. The reasons for this are unclear, but it has been suggested that between-country differences in diet (including food-fortification policies) and vitamin D supplement use are contributing factors^(21)^.

Using harmonised data on 25(OH)D from more than 80 000 participants in the Biomarkers for Cardiovascular Risk Assessment in Europe (BiomarCaRE) project, we conducted the largest study to date on the association between geographical latitude and 25(OH)D status in Europe.

## Subjects and methods

### Study population

The present study is based on data from the BiomarCaRE project (www.biomarcare.eu) – details of which have been published elsewhere^(24)^ – which, in turn, is based on the Monitoring of Trends and Determinants in Cardiovascular Disease (MONICA) Risk, Genetics, Archiving, and Monograph (MORGAM) project^(25,26)^. The MORGAM/BiomarCaRE Data Centre in Helsinki, Finland, harmonises and stores individual data from a large number of population-based European cohorts, and the BiomarCaRE laboratory in Hamburg (previously in Mainz), Germany, analyses and stores blood samples from several hundred thousand cohort participants.

**Fig. 1.**
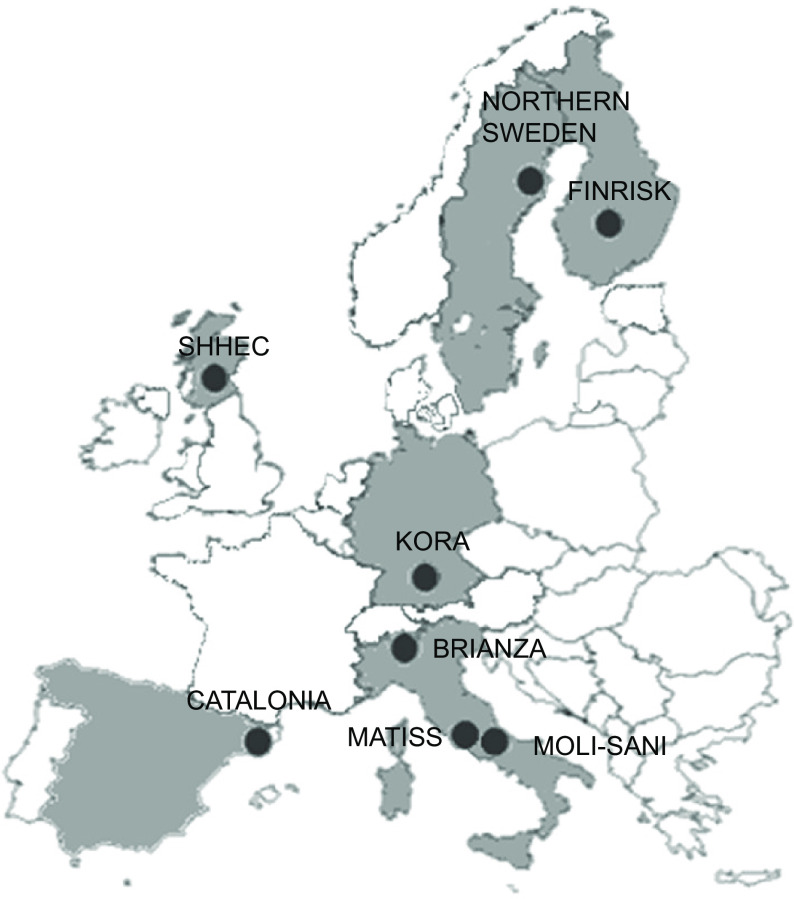
Approximate geographical location of the included cohorts. SHHEC, Scottish Heart Health Extended Cohort; KORA, Cooperative Health Research in the Region of Augsburg; MATISS, Malattie Aterosclerotiche Istituto Superiore di Sanità.

A total of eight population-based cohorts (including 81 084 participants, of whom 3682 were examined more than once) from six European countries (Sweden, Finland, Scotland, Germany, Italy and Spain) had blood samples sent to the BiomarCaRE laboratory for 25(OH)D measurement and were included in the present study (Online Resource 1). The individual cohorts were: (1) MONICA Northern Sweden (Sweden; examination period 1986, 1990, 1994, 1999 (which also included re-examinations of the surveys in 1986–1994), 2004, 2009 and 2014); (2) FINRISK 1997 (Finland; examination period 1997); (3) Scottish Heart Health Extended Cohort (SHHEC) (Scotland; examination period 1984–1987, 1989, 1992 and 1995); (4) MONICA/Cooperative Health Research in the Region of Augsburg (KORA) (Germany; examination period 1994–1995 and 1999–2001); (5) MONICA Brianza (Italy; examination period 1986, 1990 and 1993); (6) Malattie Aterosclerotiche Istituto Superiore di Sanità (Italy; examination period 1993–1996); (7) Moli-sani (Italy; examination period 2005–2010) and (8) MONICA-Catalonia (Spain; examination period 1986–1988 and 1990–1992). Most cohorts examined participants throughout the calendar year (except for the Swedish and Finnish cohort, which examined participants almost exclusively in January to April) and almost all participants were assessed by serum samples (except for 4·4 % in the Swedish cohort, for whom only plasma samples were available). Each cohort was deemed representative of the population of interest (with participation rates ranging from 60 to 81 %). Further details of the cohorts are presented in Online Resource 2, with their approximate geographical location shown in Fig. 1.

### Ethical approval

This study was conducted according to the guidelines laid down in the Declaration of Helsinki, and all procedures involving human subjects were approved by relevant local ethical review boards. Written informed consent was obtained from all participants. The recommendations of the Strengthening the Reporting of Observational Studies in Epidemiology initiative were followed whenever applicable^(27)^.

### Assessment of geographical latitude and other variables

Data on participant (i.e. sex, age and date of blood sampling) and blood sample characteristics (i.e. type of sampling material (serum or plasma) and date of analysis of 25(OH)D) were obtained from the MORGAM/BiomarCaRE Data Centre in Helsinki, Finland. Data on geographical latitude were based on the location of the largest city in each cohort’s recruitment area and retrieved from Google Maps (www.google.com/maps), except for in the SHHEC where district-level data on geographical latitude were available. For cohorts that had recruited participants over a large geographical area (i.e. MONICA Northern Sweden, FINRISK 1997 and SHHEC), the participants were classified into subcohorts according to the recruitment area or district area. The estimated geographical latitudes for the included cohorts are presented in detail in Online Resource 3. In brief, along a north-to-south axis, the following geographical latitudes were applied for each country: Sweden (65·6–63·8°N), Finland (65·0–60·2°N), Scotland (58·2–55·1°N), Germany (48·4°N), Italy (45·6–41·5°N) and Spain (41·5°N).

### Assessment of 25-hydroxyvitamin D status

Serum or plasma analyses of 25(OH)D were conducted between 2009 and 2018 at the BiomarCaRE laboratory in Germany (located in Mainz up until 2012, thereafter in Hamburg) using a 1-step immunoassay on the Abbott ARCHITECT i2000 (Abbott Diagnostics)^(28)^. Previous research has shown that serum and plasma measurements of 25(OH)D are highly similar^(29)^. The cohort-specific years of analysis and intra-assay and inter-assay coefficients of variation are shown in Online Resource 4. No evidence of laboratory drift was observed during the study period (data not shown).

In a subgroup of the MONICA Northern Sweden cohort (*n* 1522), the 25(OH)D status estimated from the Abbott ARCHITECT has been compared with that estimated from a HPLC-MS/MS (traceable to the National Institute of Standards and Technology’s Standard Reference Material 972)^(30,31)^, which is considered a more accurate method for 25(OH)D analyses^(32)^. Although the measurement methods had a good correlation in terms of rank (Spearman’s coefficient (*r*) = 0·82–0·91, irrespective of sex and age group), the 25(OH)D concentrations were on average 8·4 ng/ml lower when measured with the Abbott ARCHITECT.

### Statistical analysis

A total of 81 084 participants and 84 766 blood samples were included for analysis. Data on 25(OH)D status were missing for 3832 blood samples (4·5 %, ranging from 1·0 % in the Swedish cohort to 11·3 % in the Scottish cohort). The main reasons for missing data were too small blood sample volumes (for the Scottish, German, Italian and Spanish cohorts) and technical issues during the laboratory procedure (for the Swedish and Finnish cohort). (See Online Resource 4 for cohort-specific percentages of missing data and reasons for missing data.) Missing data were handled using multiple imputation by chained equations (10 data sets were created and combined using Rubin’s rule)^(33)^, in which sex, age, calendar year of sampling, calendar week of sampling, type of sampling material and cohort were included as regular variables. There was no evidence of violation of the missing at random assumption (data not shown). In a sensitivity analysis, we repeated the analyses using complete case data.

To study the exposure–outcome association in detail, we used quantile regression models and calculated percentile values (1st–99th) of 25(OH)D status according to country (ordered by decreasing geographical latitude: Sweden (comparator), Finland, Scotland, Germany, Italy and Spain) and geographical latitude (continuous, °N). Geographical latitude was modelled as a continuous variable assuming linearity (i.e. a constant change in 25(OH)D concentration for each unit of change in geographical latitude). To relax this assumption, we used 4-knot restricted cubic splines in a continuous analysis (knots at 65·0, 55·8, 48·4 and 41·6°N)^(34)^ and also performed a separate categorical analysis (using the northernmost subcohort (at 65·6°N) as comparator). A test for non-linearity in the restricted cubic spline model was conducted by testing the coefficients of the second and third spline transformation jointly equal to zero. In a sensitivity analysis, we repeated the analyses using mean regression models.

Multivariable quantile regression models were adjusted for sex, age (continuous, years), decade of sampling (1980s, 1990s, 2000s), calendar week of sampling (continuous, calendar weeks) and time from sampling to analysis (continuous, years). Since there was evidence of non-linear associations between 25(OH)D status and age (inverse J curve), calendar week of sampling (seasonal curve, as shown in Online Resource 5) and time from sampling to analysis (right-tilted L curve), we modelled these covariates using 4-knot restricted cubic splines (knots at the 5th, 35th, 65th and 95th percentile). Varying the number of knots (to 3 or 5 knots) had negligible influence on the results (data not shown).

Separate quantile regression models by (1) sex, (2) age (<51 (median), ≥51 years), (3) decade of sampling (1980s, 1990s, 2000s) and (4) season of sampling (winter (December to February), spring (March to May), summer (June to August), fall (September to November)) were performed as subgroup analyses. Since a proportion of the Swedish cohort had been examined more than once (treated as independent observations in the main analysis; *n* 3682) and/or with plasma instead of serum (*n* 687), we also performed a sensitivity analysis by restricting the quantile regression model to first-time serum blood samples (*n* 80 397).

Statistical significance was set at a two-sided *P*-value less than 0·05. Analyses were performed using Stata version 14 (StataCorp LP).

## Results

A total of 81 084 participants (48·9 % men; median age 50·8 years), who contributed with 84 766 blood samples during the 1980s (21·8 %), 1990s (39·5 %) and 2000s (38·8 %), were included for analysis. Forty point two percentage were from Italy (*n* 32 572), 18·4 % from Scotland (*n* 14 902), 14·8 % from Sweden (*n* 11 973, of whom 3682 had been examined more than once), 10·4 % from Germany (*n* 8393), 9·9 % from Finland (*n* 8002) and 6·5 % from Spain (*n* 5242). Blood samples were more often drawn in winter (December to February; 31·3 %) and spring (March to May; 34·7 %) than in summer (June to August; 14·4 %) and fall (September to November; 19·8 %).

Participant and blood sample characteristics by country are shown in Table 1. (Nota bene: the Italian study population was recruited from three separate cohorts, for which details are given in Online Resource 6.) Apart from the Italian and Spanish cohort (with the largest and smallest percentage of women (53·2 *v*. 45·7 %) and the highest and lowest median age (53·1 *v*. 45·7 years), respectively), the cohorts’ sex and median age structures were quite homogenous (female sex 49·8–50·9 %; median age 48·4–50·2 years). In contrast, there was a large between-cohort difference in the time period of sampling and, subsequently, in the time from sampling to analysis (as an example, the overwhelming majority of Scottish and Italian participants were sampled in the 1980s and 2000s, respectively, while all Finnish participants were sampled in the 1990s). Most cohorts had sampled participants throughout the calendar year, except for the Swedish and Finnish cohort, which had sampled participants almost exclusively in winter and spring. A small percentage of Swedish participants had been sampled with plasma instead of serum.

**Table 1.** Baseline characteristics of the study population (*n* 81 084, including 84 766 observations) by country (Percentages unless otherwise specified)

	Country (ordered by decreasing geographical latitude)					
	Sweden	Finland	Scotland	Germany	Italy*	Spain
No. of participants	11 973	8002	14 902	8393	32 572	5242
Participant characteristics†						
Age (years)						
Median	51·2	48·4	49·8	50·2	53·1	45·7
Range	24·1, 79·3	24·2, 74·3	25·2, 75·8	24·6, 75·5	20·6, 98·7	24·8, 67·8
Male sex	49·1	50·0	50·2	49·7	46·8	54·3
Decade of sampling						
1980s	10·2	–	77·9	–	8·5	47·0
1990s	57·9	100	22·1	57·1	17·0	53·0
2000s	31·9	–	–	42·9	74·5	–
Season of sampling						
Winter (December to February)	52·9	55·6	15·6	32·1	22·9	22·2
Spring (March to May)	47·1	44·4	33·3	31·1	28·7	29·4
Summer (June to August)‡	–	–	27·6	8·6	19·8	18·1
Fall (September to November)‡	–	–	23·5	28·2	28·6	30·3
Blood sample characteristics†						
Time to analysis (years)						
Median	18·3	15·9	24·3	19·8	7·7	25·7
Range	3·5, 30·8	15·3, 16·1	15·9, 27·2	13·9, 20·6	4·5, 28·8	24·1, 30·3
Type of sampling material						
Serum	95·6	100	100	100	100	100
Plasma	4·4	–	–	–	–	–

* The Italian study population was recruited from three separate cohorts, for which details are given in Online Resource 6.

† Based on the total number of observations in each cohort (3682 participants in the Swedish cohort were examined more than once, leading to a total of 15 655 observations).

‡ Three participants in the Swedish cohort and one participant in the Finnish cohort were sampled in summer or fall.

**Fig. 2.**
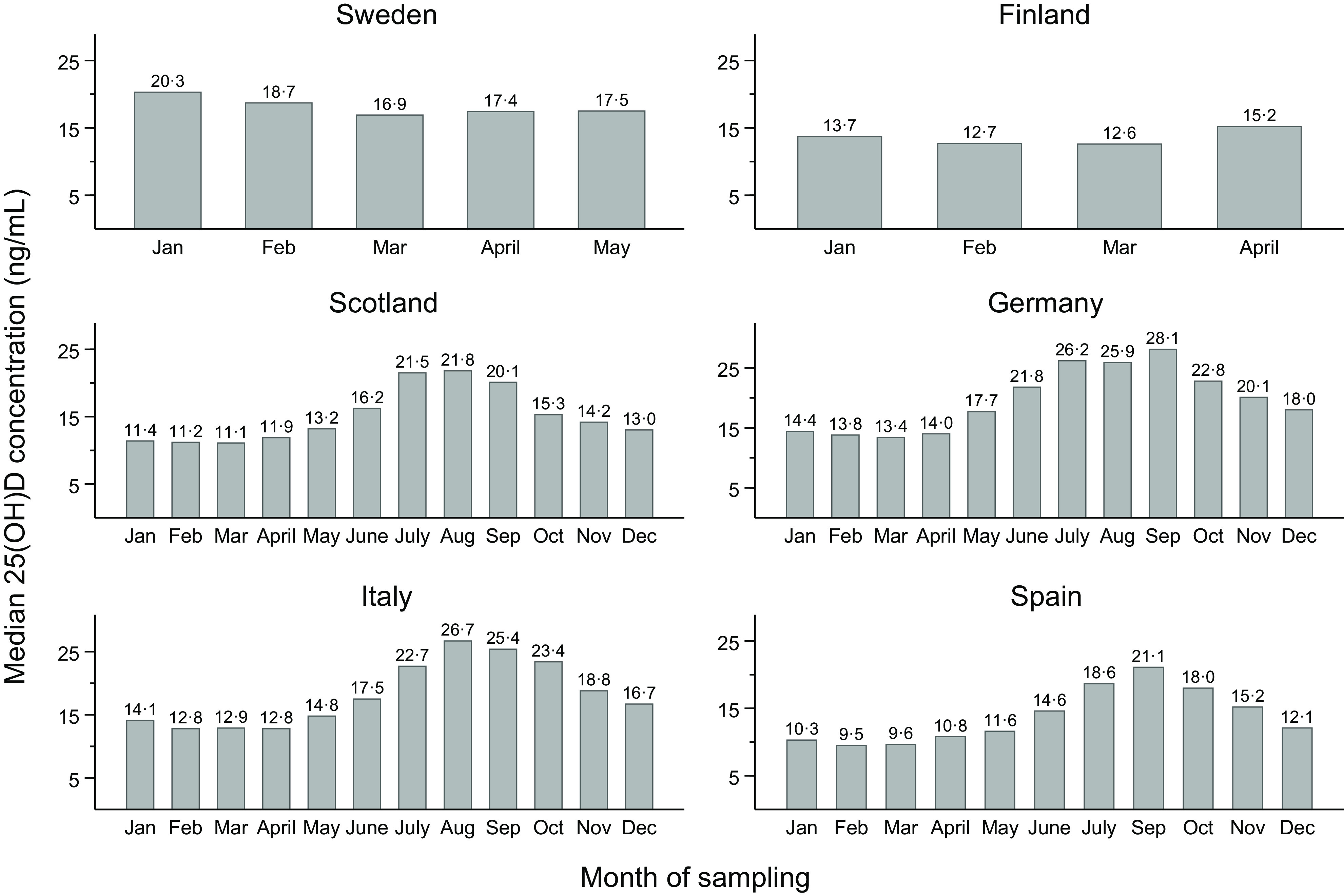
Observed monthly variation of 25-hydroxyvitamin D (25(OH)D) status in the study population with complete data on 25(OH)D (*n* 77 320, including 80 934 observations) by country. The day length on 1st January and 1st July, respectively (using 2019 as an example year), was 4:33 and 20:37 h in Sweden (Umeå), 5:59 and 18:47 h in Finland (Helsinki), 7:05 and 17:30 h in Scotland (Edinburgh), 8:24 and 16:02 h in Germany (Augsburg), 8:46 and 15:38 h in Italy (Monza), and 9:15 and 15:07 h in Spain (Sabadell). (Information on day length was derived from www.timeanddate.com).

The median 25(OH)D concentration was 16·4 ng/ml in the entire study population, 15·5 ng/ml in the female population and 17·3 ng/ml in the male population. As expected, there was a strong seasonal variation of 25(OH)D status (Fig. 2). In cohorts that had sampled participants throughout the calendar year, the highest and lowest median concentrations were observed in August to September and February to March, respectively, with an absolute (relative) peak-to-minimum variation of 10·7 ng/ml (96 %) in Scotland, 14·7 ng/ml (110 %) in Germany, 13·9 ng/ml (110 %) in Italy and 11·6 ng/ml (122 %) in Spain. The difference in 25(OH)D status according to the decade of sampling is presented in Online Resource 7. There were indications of higher 25(OH)D concentrations over time, with an absolute (relative) 2000s-to-1980s variation of 1·4 ng/ml (10 %) during winter, 2·0 ng/ml (16 %) during spring, 3·1 ng/ml (17 %) during summer and 5·4 ng/ml (31 %) during fall.

**Fig. 3.**
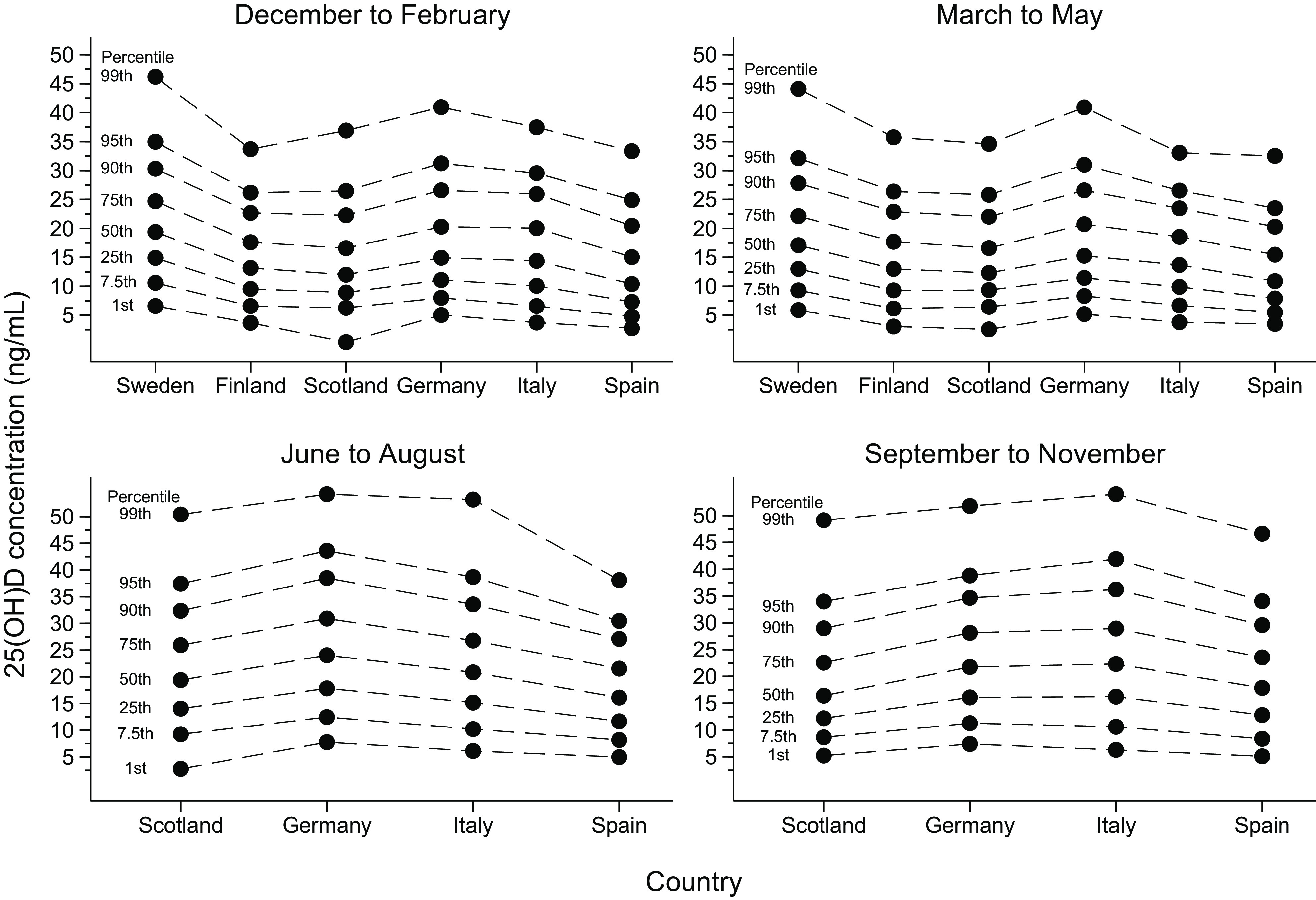
Percentile distribution of 25-hydroxyvitamin D (25(OH)D) status in the study population (*n* 81 084, including 84 766 observations) by country (ordered by decreasing geographical latitude) and season of sampling (winter, spring, summer and fall) and based on multiple imputed data sets (*n* 10). The *solid dots* represent unadjusted percentile values for each country. The *dashed lines* are added to facilitate between-country comparisons of each percentile.

Unadjusted percentile values of 25(OH)D status by country are shown in Fig. 3. In the multivariable model, the ordering of countries by 25(OH)D status (from highest to lowest) was as follows up until the median percentile: Sweden (at 65·6–63·8°N), Germany (at 48·4°N), Finland (at 65·0–60·2°N), Italy (at 45·6–41·5°N), Scotland (at 58·2–55·1°N) and Spain (at 41·5°N) (Table 2). From the 75th percentile and upwards, the Finnish cohort had higher values than the German cohort. As an example, using the Swedish cohort as comparator, the median (25th, 75th percentile) 25(OH)D concentration was 3·03 (2·36, 3·75) ng/ml lower in the German cohort, 3·28 (2·96, 3·48) ng/ml lower in the Finnish cohort, 5·41 (4·61, 6·17) ng/ml lower in the Italian cohort, 6·54 (5·27, 7·49) ng/ml lower in the Scottish cohort and 9·28 (7·65, 10·59) ng/ml lower in the Spanish cohort (*P*-value < 0·001 for all comparisons). The ordering of countries was highly consistent, although with some between-country differences in the magnitude of the exposure–outcome association, in subgroup analyses by sex, age, and decade and season of sampling (Table 3). Likewise, the results were similar in the sensitivity analysis restricted to first-time serum blood samples (median difference (ng/ml) compared with Sweden: Germany −2·73, Finland −2·32, Italy −5·16, Scotland −6·12 and Spain −9·23; *P*-value < 0·001 for all comparisons) and in the sensitivity analysis based on the complete case data (Online Resource 8). The interpretation of the results did not change in the sensitivity analysis using mean regression (data not shown).

**Table 2.** Differences in 25(OH)D status in the study population (*n* 81 084, including 84 766 observations) by country and based on multiple imputed data sets (*n* 10)

25(OH)D status (ng/ml)	Country (ordered by decreasing geographical latitude)											
	Sweden		Finland		Scotland		Germany		Italy		Spain	
	Percentile difference*	95 % CI	Percentile difference*	95 % CI	Percentile difference*	95 % CI	Percentile difference*	95 % CI	Percentile difference*	95 % CI	Percentile difference*	95 % CI
5^th^	Comp.	–	−2·17	−2·50, –1·84	−4·15	−4·46, –3·84	−1·33	−1·62, –1·04	−3·35	−3·57, –3·14	−5·59	−5·86, –5·33
10^th^	Comp.	–	−2·46	−2·77, –2·14	−4·29	−4·57, –4·01	−1·43	−1·72, –1·14	−3·75	−3·95, –3·56	−6·31	−6·57, –6·05
25^th^	Comp.	–	−2·96	−3·25, –2·67	−5·27	−5·51, –5·03	−2·36	−2·62, –2·10	−4·61	−4·79, –4·43	−7·65	−7·88, –7·41
50^th^	Comp.	–	−3·28	−3·65, –2·91	−6·54	−6·86, –6·23	−3·03	−3·34, –2·73	−5·41	−5·62, –5·19	−9·28	−9·59, –8·97
75^th^	Comp.	–	−3·48	−3·95, –3·00	−7·49	−7·87, –7·11	−3·75	−4·13, –3·36	−6·17	−6·45, –5·88	−10·59	−11·01, –10·18
90^th^	Comp.	–	−3·66	−4·35, –2·97	−8·12	−8·77, –7·48	−4·11	−4·68, –3·53	−7·22	−7·65, –6·80	−12·12	−12·72, –11·52
95^th^	Comp.	–	−4·21	−5·21, –3·22	−8·59	−9·50, –7·68	−4·69	−5·57, –3·81	−8·24	−8·92, –7·56	−12·98	−13·87, –12·09

25(OH)D, 25-hydroxyvitamin D; Comp., comparator.

* Estimated from quantile regression models adjusted for sex, age (continuous using 4-knot restricted cubic splines (4-RCS), years), calendar week of sampling (continuous using 4-RCS, calendar weeks), decade of sampling (1980s, 1990s, 2000s) and time from sampling to analysis (continuous using 4-RCS, years).

**Table 3.** Subgroup analyses of differences in 25(OH)D status in the study population (*n* 81 084, including 84 766 observations) by country and based on multiple imputed data sets (*n* 10)

25(OH)D status (ng/ml)	Country (ordered by decreasing geographical latitude)											
	Sweden		Finland		Scotland		Germany		Italy		Spain	
	Median difference*	95 % CI	Median difference*	95 % CI	Median difference*	95 % CI	Median difference*	95 % CI	Median difference*	95 % CI	Median difference*	95 % CI
By sex												
Men	Comp.	–	−2·22	−2·74, –1·70	−5·65	−6·09, –5·20	−2·11	−2·53, –1·69	−3·59	−3·89, –3·28	−8·46	−9·06, –8·23
Women	Comp.	–	−4·23	−4·73, –3·74	−7·37	−7·78, –6·96	−4·04	−4·44, –3·64	−6·90	−7·19, –6·60	−9·87	−10·27, –9·47
By median age												
<51 years	Comp.	–	−3·5	−4·02, –3·08	−5·77	−6·19, –5·36	−1·59	−1·99, –1·18	−3·46	−3·76, –3·15	−8·37	−8·73, –8·01
≥51 years	Comp.	–	−1·94	−2·44, –1·43	−6·80	−7·22, –6·38	−4·38	−4·75, –4·01	−7·33	−7·61, –7·06	−10·09	−10·49, –9·68
By decade of sampling												
1980s	Comp.	–	–	–	−6·43	−6·91, –5·94	–	–	−3·71	−4·37, –3·06	−7·65	−8·20, –7·10
1990s	Comp.	–	−5·44	−5·70, –5·19	−7·05	−7·45, –6·64	−5·19	−5·54, –4·85	−6·67	−7·02, –6·32	−9·07	−9·42, –8·72
2000s	Comp.	–	–	–	–	–	−2·57	−2·96, –2·18	−3·92	−4·20, –3·64	–	–
By season of sampling†,‡												
Winter	7·77	7·20, 8·35	2·77	1·89, 3·65	Comp.	–	3·67	3·09, 4·25	1·55	0·96, 2·14	−2·06	−2·76, –1·36
Spring	4·95	4·58, 5·32	2·91	2·33, 3·50	Comp.	–	2·89	2·38, 3·40	0·50	0·11, 0·88	−2·58	−3·05, –2·11
Summer	–	–	–	–	Comp.	–	5·18	3·65, 6·72	0·31	−0·40, 1·03	−2·52	−3·59, –1·44
Fall	–	–	–	–	Comp.	–	6·73	5·91, 7·56	3·75	3·02, 4·49	−3·25	−4·40, –2·10

25(OH)D, 25-hydroxyvitamin D; Comp., comparator.

* Estimated from quantile regression models adjusted for sex, age (continuous using 4-knot restricted cubic splines (4-RCS), years), calendar week of sampling (continuous using 4-RCS, calendar weeks), decade of sampling (1980s, 1990s, 2000s) and time from sampling to analysis (continuous using 4-RCS, years).

† Only three participants in the Swedish cohort and one participant in the Finnish cohort were sampled in summer or fall; therefore, to facilitate between-country comparisons across all subgroups, Scotland was used as the comparator group.

‡ Adjusted for the 3 months in each season of sampling (as a categorical variable).

**Fig. 4.**
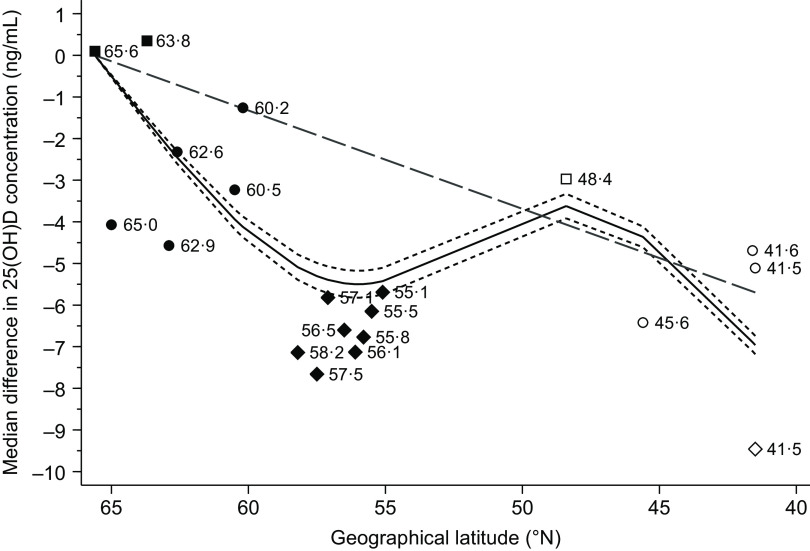
Differences in 25-hydroxyvitamin D (25(OH)D) status in the study population (*n* 81 084, including 84 766 observations) by geographical latitude and based on multiple imputed data sets (*n* 10). The *solid line* represents median differences and the *short dashed lines* represent 95 % CI from a multivariable 4-knot restricted cubic spline model (knots at 65·0, 55·8, 48·4 and 41·6°N). The *long dashed line* represents median differences from a multivariable linear dose–response model. The *symbols* represent median differences from a multivariable categorical model. The *numbers* adjacent to the symbols represent the geographical latitude of each cohort or subcohort. The estimates were derived from quantile regression models that were adjusted for the same covariates as the multivariable model in Table 2. The comparator value was set to the northernmost subcohort at geographical latitude 65·6°N. 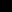, Sweden; 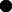, Finland; 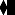, Scotland; 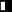, Germany; 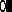, Italy; 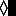, Spain.

In the analysis of geographical latitude as a continuous variable, there was a positive association between geographical latitude and 25(OH)D status (median increase of 0·23 ng/ml for each 1-unit increase in geographical latitude (95 % CI 0·22, 0·24; *P*-value < 0·001)). However, as shown in Fig. 4, this linear dose–response model captured the shape of the exposure–outcome association very poorly compared with the restricted cubic spline model (*P*-value < 0·001 for non-linearity) and, especially, the categorical model. In countries with multiple recruitment areas, the within-country median difference in 25(OH)D concentration ranged from 0·35 ng/ml in Sweden to 2·81 ng/ml in Finland (based on the categorical exposure model).

## Discussion

In this cross-sectional study, which included more than 80 000 participants from six European countries, we observed a north-to-south gradient of 25(OH)D status (although not linear in its shape), with higher percentile values in the northernmost countries (i.e. Sweden and Finland) compared with the southernmost countries (i.e. Spain and Italy). These findings were highly consistent in subgroup analyses by sex, age, and decade and season of sampling.

Several other studies with harmonised or standardised data on 25(OH)D have reported on a north-to-south gradient in Europe^(16–23)^. In the EURONUT-SENECA study (*n* 824), men and women from Norway and Denmark had higher mean concentrations of 25(OH)D than their counterparts in Spain and Italy^(16)^. Similar findings were observed in three randomised clinical trials of postmenopausal women (*n* 997–3195), where the mean concentrations of 25(OH)D were higher in northern Europe than in southern Europe^(17–19)^. Deleskog *et al*. also reported on a positive association between geographical latitude and 25(OH)D status in a cohort of 3430 European men and women^(20)^. In the ODIN project, which combined and standardised 25(OH)D data from eighteen European studies (*n* 55 844), the mean concentrations of 25(OH)D were higher in Norway and Iceland – but slightly lower in Finland – compared with Greece^(21)^. Likewise, the mean concentrations of 25(OH)D were higher in Norwegian participants than in south-European participants in the European Eye study (*n* 4495)^(22)^. Finally, in the European Prospective Investigation into Cancer and Nutrition-Interact case-cohort study (*n* 22 651), the authors observed a positive association between geographical latitude and 25(OH)D status^(23)^.

Sunlight is an important determinant of 25(OH)D status, as exemplified by the strong seasonal variation in our data (the median concentration in the pooled population was 9·8 ng/ml lower in March than in August and September). However, based on the available literature of a north-to-south gradient, it is questionable if sunlight should be viewed as *the most important* determinant of 25(OH)D status in today’s society^(13)^, at least from a European perspective. In analogy, it may not be correct to view north-European populations as more at risk for vitamin D insufficiency than other European populations, despite the fact that the cutaneous synthesis of 25(OH)D is virtually undetectable during winter at geographical latitudes above 50°N^(14)^.

Other factors that have a large influence on 25(OH)D status are the consumption of vitamin D-rich food items (e.g. fatty fish and shellfish) and vitamin D-fortified food items (e.g. milk and margarine) as well as the use of vitamin D-containing supplements^(13)^. In the European Prospective Investigation into Cancer and Nutrition calibration study, which used a standardised FFQ and was based on data from 1995 to 2000 (*n* 36 034), it was observed that north-Europeans had a higher food intake of vitamin D than south-Europeans^(35)^. The highest intakes were seen in the Swedish participants, most likely due to Sweden’s long tradition of voluntary – and since 2007 mandatory – vitamin D fortification of milk, margarine and cooking fats^(30)^. Interestingly enough, given that the Swedish cohort already had the highest 25(OH)D concentrations in our data, the vitamin D food-fortification policy in Sweden was expanded in 2018, so that it now includes more food items and dictates a higher quantity of vitamin D in each food item (Swedish National Food Agency, personal communication, 2019). At the time when the Finnish cohort was examined (1997), the Finnish legislation only allowed for voluntary vitamin D fortification of certain milk and margarine products by permission (Finnish Food Authority, personal communication, 2020). Since then, the Finnish Food Authority has started to recommend vitamin D fortification of all milk and margarine products, to which most companies have complied and that has led to increased 25(OH)D concentrations in the Finnish population^(36)^. In the other countries that were included in our study, vitamin D-fortified products are commercially available, but its consumption is not specifically advised and there are no mandatory food-fortification policies (with the exception of margarine products in Scotland)^(37)^. With respect to supplement use, it was observed in the previously mentioned European Prospective Investigation into Cancer and Nutrition calibration study that Swedish participants used supplements four to five times as often as Italian and Spanish participants^(38)^. The Swedish participants were also more likely to use supplements that contained vitamin D. A potential increase in the use of vitamin D supplements in Europe over time (as noted in the USA^(39)^) could, in turn, be a partial explanation to the seemingly higher 25(OH)D concentrations in the 2000s compared with the 1980s in our data.

In addition to a large sample size that was recruited from six European countries (with geographical latitudes ranging from 65·6 to 41·5°N), the main strength of the current study was the use of harmonised data on 25(OH)D (i.e. analysed in the same lab and with the same assay method). As often discussed in the past, comparative studies on 25(OH)D status can be severely hampered by variations in assay method^(40–42)^. Other strengths were the use of multiple percentile values to fully describe the exposure–outcome association as well as the fact that blood metabolites of 25(OH)D have been shown to be robust to handling^(43)^, storage duration^(44)^ and multiple freeze–thaw cycles^(45)^.

A number of limitations of the current study must be mentioned. Firstly, we had no individual data on geographical latitude and instead used an ecological assignment (i.e. the geographical latitude of the largest city in each recruitment area, except for in the SHHEC where district-level data were available). While the ecological assignment did not affect the between-country comparisons, it led to clustering of participants within recruitment areas and hindered a more detailed analysis of geographical latitude as a continuous variable. Secondly, we used geographical latitude as a proxy for sunlight incidence, assuming a direct correlation between geographical latitude and the hours and quality of sunlight. It is, however, possible that the months preceding the blood sampling could have had unusually many (or few) total hours of sunlight in a specific region, which, in turn, could have affected the within-country comparisons and the between-country comparisons for recruitment areas located close to each other (i.e. Sweden *v*. Finland and Italy *v*. Spain). With that said, it is unlikely that local weather deviations should have had a big impact on the more extreme comparisons, such as Sweden (at 65·6–63·8°N) compared with Spain (at 41·5°N). In addition, we had no data on the participants’ sun behaviour (e.g. actual time spent in the sunlight, avoidance of sunlight during the warmest hours and type of clothing used in the sunlight), which is likely to have differed between countries. Thirdly, the data on 25(OH)D status in the Swedish and Finnish cohorts were restricted to participants sampled in winter and spring. As consequence, we had to assume in the statistical model that the seasonal variation of 25(OH)D status was the same in all countries, which, based on the observed data (where the country-specific seasonal variation ranged from 10·7 to 14·7 ng/ml), might have skewed the multivariable-adjusted estimates to a certain degree. However, the relative seasonal variation in the Swedish cohort had had to be less than 66 % (median value) and 60 % (mean value) in order to not be ranked first in peak 25(OH)D status, which is lower than the seasonal variation recently reported in a Swedish study (mean variation of 73 % between February and July)^(46)^. A proportion of the Swedish participants had also been sampled more than once (and treated as independent observations), leading to potential bias in the quantile regression model. However, the results were highly similar in the sensitivity analysis restricted to first-time blood samples. Fourthly, there was a rather large variability in the between-cohort percentages of missing 25(OH)D data (ranging from 1·0 to 11·3 %). In cohorts with more than 3 % missing data, the main reason for missingness was that the blood sample volumes were too small due to previous analyses at the BiomarCaRE laboratory. However, bias by missing data should have been kept to a minimum with the use of multiple imputation techniques^(47)^. Fifthly, we had no data on – and could not adjust the analyses for – food and beverage consumption or use of vitamin D supplements. Sixthly, even though all of the included studies were population based in design, we cannot with certainty say that our findings are generalisable to populations outside of the recruitment areas or to the current point in time (especially since the Swedish and Finnish food-fortification policies have changed in recent years). A final limitation was that the 25(OH)D status was estimated with a 1-step immunoassay (Abbott ARCHITECT) and not a HPLC-MS/MS calibrated to a standard reference measurement procedure^(32)^. Our research group has previously validated the Abbott ARCHITECT against a HPLC-MS/MS (traceable to the National Institute of Standards and Technology’s Standard Reference Material 972) in a subgroup of the MONICA Northern Sweden cohort^(30,31)^, and we observed a good correlation (in terms of rank, *r* = 0·82–0·91) but a general underestimation (on average 8·4 ng/ml) of 25(OH)D concentrations. As such, the absolute percentile values in our study should be interpreted with caution and not used for the classification of vitamin D deficiency^(12)^ (to do so, a Vitamin D Standardisation Program protocol had been required^(15)^). However, due to the harmonised measurement, there is no reason to suspect a substantial variability in the underestimation of 25(OH)D status by country or cohort, meaning that the relative differences in percentile values should be valid.

In conclusion, in the largest study to date on the association between geographical latitude and 25(OH)D status in Europe, we confirmed the previous observation of a north-to-south gradient, with higher percentile values among north-Europeans than south-Europeans. These findings indicate that other factors are as, if not more, important as sunlight for 25(OH)D status in today’s European society. Future studies are needed to understand the underlying reasons for a north-to-south gradient of 25(OH)D status in Europe.

## Supplementary material

For supplementary material accompanying this paper visit https://doi.org/10.1017/S0007114521005080. click here to view supplementary material
