# Supplementary material for: Influence of geographical latitude on vitamin D status: cross-sectional results from the BiomarCaRE consortium
Source: Br J Nutr. 2021 Dec 22;128(11):2208–18. doi: 10.1017/S0007114521005080 (PMC9661368; doi:10.1017/S0007114521005080)
Supplement: Supplementary file 1 [file S0007114521005080sup001.pdf]

Influence of geographical latitude on vitamin D status: cross-sectional results from the BiomarCaRE consortium

Author information: Viktor Oskarsson, Mats Eliasson, Veikko Salomaa, Jaakko Reinikainen, Satu Männistö, Luigi Palmieri, Chiara Donfrancesco, Susana Sans, Simona Costanzo, Giovanni de Gaetano, Licia Iacoviello, Giovanni Veronesi, Marco M Ferrario, Teresa Padro, Barbara Thorand, Cornelia Huth, Tanja Zeller, Stefan Blankenberg, Annie S Anderson, Hugh Tunstall-Pedoe, Kari Kuulasmaa, and Stefan Söderberg; on behalf of the BiomarCaRE investigators

Corresponding author: Viktor Oskarsson, Piteå Research Unit, Department of Public Health and Clinical Medicine, Umeå University, 901 87 Umeå, Sweden (e-mail: viktor.oskarsson @umu.se; phone: +46 90 785 00 00; fax: +46 90 137 633; ORCID iD: 0000-0002-2936-2895)

Online Resource 1 Participant flow chart of the included cohorts

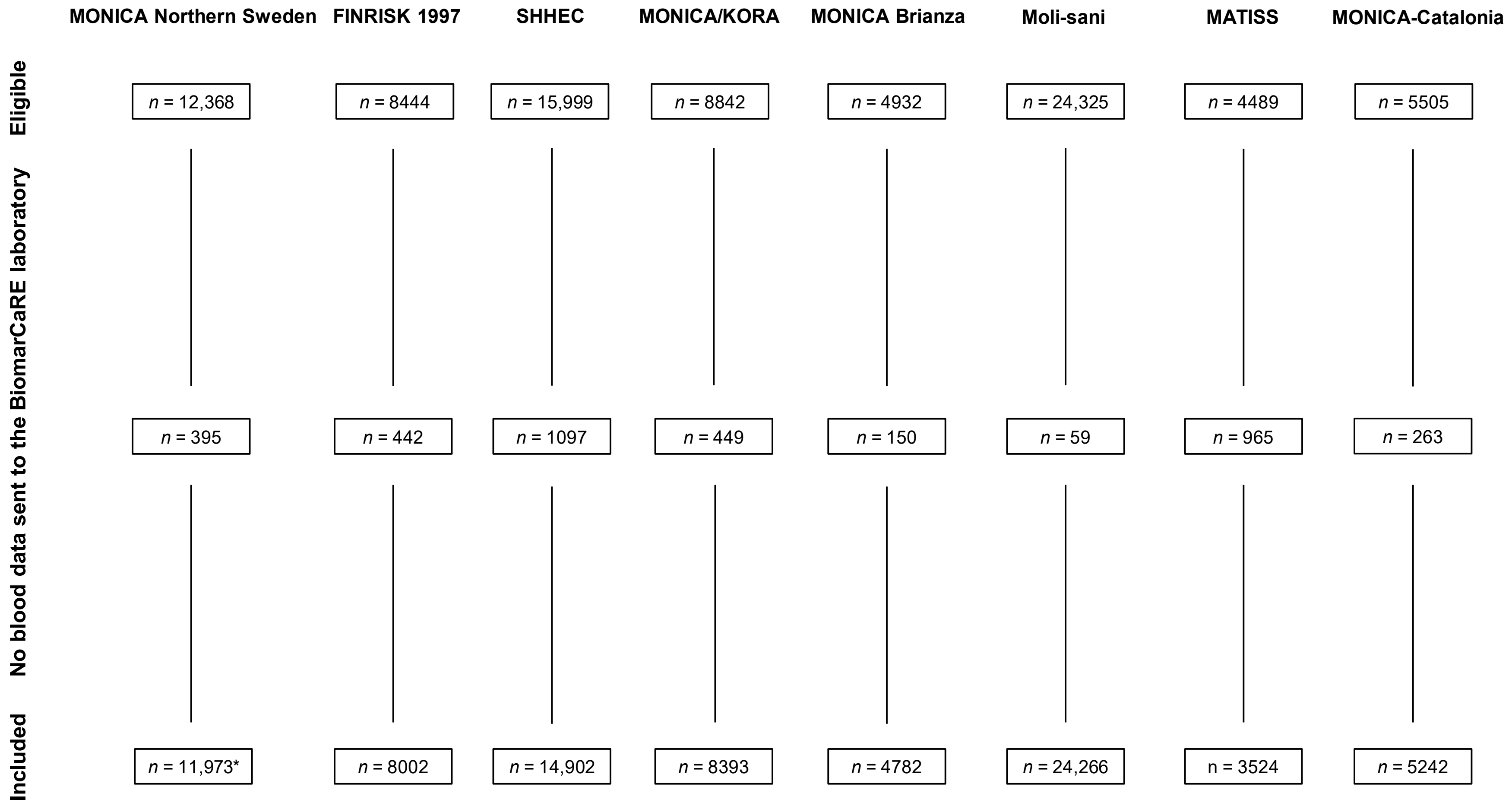

Abbreviations: MONICA, Monitoring of Trends and Determinants in Cardiovascular disease; SHHEC, Scottish Heart Health Extended Cohort; KORA, Cooperative Health Research in the Region of Augsburg; MATISS, Malattie Aterosclerotiche Istituto Superiore di Sanità; BiomarCaRE, Biomarkers for Cardiovascular Risk Assessment in Europe

* Three thousand six hundred eighty-two participants in the Swedish cohort were examined more than once, leading to a total of 15,655 observations

Online Resource 2 Overview and description of the included cohorts

Cohort	Country	Description
MONICA Northern Sweden	Sweden	The Northern Sweden MONICA study consists of 7 population-based surveys (in 1986, 1990, 1994, 1999, 2004, 2009, and 2014) in the 2 northernmost counties of Sweden (i.e., Norrbotten and Västerbotten). Individuals were randomly selected from population registers and stratified for age (25 to 64 years in 1986 and 1990; 25 to 74 years in 1994 to 2014) and sex. In 1999, all individuals in the 3 previous surveys were re-invited for repeated measurements. Overall, the participation rate ranged from 63 to 81%. *Details: www.thl.fi/publications/morgam/cohorts/full/sweden/swe-nswa.htm*
FINRISK 1997	Finland	The FINRISK 1997 study is a population-based survey that was carried out in 5 districts of Finland, including North Karelia, northern Savo (former Kuopio), southwestern Finland, Oulu province, and the region of Helsinki and Vantaa. A stratified random sample of men and women (aged 25 to 74 years) was drawn from the national population register. The participation rate was 73%. *Details: www.thl.fi/publications/morgam/cohorts/full/finland/fin-fina.htm*
SHHEC	Scotland	The SHHEC consists of 2 overlapping studies: the Scottish Heart Health Study, which randomly recruited men and women (aged 40 to 59 years) across 22 Scottish districts in 1984 to 1987; and the Scottish MONICA study, which recruited men and women (aged 25 to 64 years) in Edinburgh (in 1986) and Glasgow (in 1986, 1989, 1992 [up to 75 years of age], and 1995) as part of the WHO MONICA Project. Overall, the participation rate ranged from 59 to 74%. *Details: www.thl.fi/publications/morgam/cohorts/full/uk/unk-sco.htm*
MONICA/KORA	Germany	The MONICA/KORA cohort consists of representative surveys from the city of Augsburg and the regions of Landkreis Augsburg and Landkreis Aichach-Friedberg in southern Germany. Municipality lists and population registers were used as sampling frames for the first and second stage of a 2-stage random sampling procedure. The second stage of sampling was stratified by sex and age. The baseline examinations in 1994 and 1995 (part of the WHO MONICA project) and in 1999 to 2001 (KORA) consisted of participants aged 25 to 74 years, with participation rates of 74 and 72%, respectively. *Details: www.thl.fi/publications/morgam/cohorts/full/germany/ger-auga.htm*

MONICA Brianza	Italy	The MONICA Brianza cohort is a prospective study of individuals in Brianza (aged 25 to 64 years), an area located between Milan and the Swiss border in northern Italy. Samples were stratified for sex and age and randomly drawn in 1986, 1990, and 1993. Overall, the participation rate ranged from 67 to 69 %. *Details: https://www.thl.fi/publications/morgam/cohorts/full/italy/ita-bria.htm*
MATISS	Italy	The MATISS study started in 1984 as a project on non-communicable diseases in central Italy. Four municipalities were involved: 3 receiving community treatment and one acting as control. Baseline (1984) and 4-year follow-up examinations were used to evaluate, in a random sample of the general population, the community treatment areas compared to the control area. In 1993 to 1996, the cohorts were re-examined and a new random sample, stratified by age (20 to 79 years) and sex, was enrolled from the residence registry (participation rate 60%). *Details: https://www.thl.fi/publications/morgam/cohorts/full/italy/ita-roma.htm*
Moli-sani	Italy	The Moli-sani cohort was established in the Molise region in southern Italy from city hall registries by a multistage sampling procedure. First, townships were sampled in major areas by cluster sampling; then, within each township, participants aged 35 years or older were selected by simple random sampling. The samples were drawn from 2005 to 2010 and the overall participation rate was 70%. *Details: https://www.thl.fi/publications/morgam/cohorts/full/italy/ita-mola.htm*
MONICA-Catalonia	Spain	The MONICA-Catalonia cohort consists of 2 population-based surveys from the central area of Catalonia and parts of the metropolitan area of Barcelona in northeastern Spain. The first stage of sampling drew a random sample from 9 municipalities with a probability proportional to the population size. In the second stage, a random sample of individuals aged 25 to 64 years from the municipal population registries were used as the sampling frame (stratified by sex and age). Baseline examinations were carried out in 1986 to 1988 (participation rate 74%) and in 1990 to 1992 (participation rate 72%). *Details: https://www.thl.fi/publications/morgam/cohorts/full/spain/spa-cata.htm*

MONICA, Monitoring of Trends and Determinants in Cardiovascular disease; SHHEC, Scottish Heart Health Extended Cohort; WHO, World Health Organization; KORA, Cooperative Health Research in the Region of Augsburg; MATISS, Malattie Aterosclerotiche Istituto Superiore di Sanità

Online Resource 3 Estimated geographical latitude for the included cohorts*

Cohort	No. of participants	Largest city	Latitude (° N)
Northern Sweden			
Norrbotten	6070	Luleå	65.6
Västerbotten	5903	Umeå	63.8
FINRISK 1997			
Oulu	1372	Oulu	65.0
Kuopio	1472	Kuopio	62.9
North Karelia	1970	Joensuu	62.6
Turku	1363	Turku	60.5
Helsinki	1825	Helsinki	60.2
SHHEC			
Highlands and Islands	374	Stornoway	58.2
Highlands and Islands	786	Inverness	57.5
North Eastern Scotland	388	Aberdeen	57.1
Eastern Scotland (1)	735	Dundee	56.5
Eastern Scotland (2)	3104	Edinburgh	56.1
South Western Scotland (1)	7144	Glasgow	55.8
South Western Scotland (2)	1960	(not available)	55.5
South Western Scotland (3)	411	(not available)	55.1
MONICA/KORA	8393	Augsburg	48.4
MONICA Brianza	4782	Monza	45.6
Moli-sani	24,266	Campobasso	41.6
MATISS	3524	Sezze	41.5
MONICA-Catalonia	5242	Sabadell	41.5

SHHEC, Scottish Heart Health Extended Cohort; MONICA, Monitoring of Trends and Determinants in Cardiovascular disease; KORA, Cooperative Health Research in the Region of Augsburg; MATISS, Malattie Aterosclerotiche Istituto Superiore di Sanità

* Data on geographical latitude were based on the location of the largest city and retrieved from Google Maps (www.google.com/maps), except for in the SHHEC where district-level data on geographical latitude were available

Online Resource 4 Details on analytical procedures and missing data of 25(OH)D by cohort

		Coefficient of variation (%)*		Missing data (%)†‡	
Cohort	Year of analysis	Intra-assay	Inter-assay	25(OH)D	Assay date
Northern Sweden	2016 to 2018	1.1 to 3.0	4.3 to 9.1	1.0	0.0
FINRISK 1997	2012 to 2013	2.8 to 4.5	6.8 to 7.2	2.7	0.8
SHHEC	2009 to 2012	5.0 to 9.5	1.4 to 8.7	11.3	10.9
MONICA/KORA	2015	2.7 to 3.5	3.7 to 4.2	4.4	0.0
MONICA Brianza	2015	4.5	2.9 to 6.2	6.1	0.0
Moli-sani	2014 to 2015	2.5 to 4.5	5.2 to 8.0	3.3	0.0
MATISS	2016	2.8 to 2.9	4.0 to 5.6	2.2	0.0
MONICA-Catalonia	2016	2.8	3.5 to 8.4	4.5	0.0

25(OH)D, 25-hydroxyvitamin D; SHHEC, Scottish Heart Health Extended Cohort; MONICA, Monitoring of Trends and Determinants in Cardiovascular disease; KORA, Cooperative Health Research in the Region of Augsburg; MATISS, Malattie Aterosclerotiche Istituto Superiore di Sanità

* Measured using a 1-step immunoassay on the Abbott ARCHITECT i2000 (Abbott Diagnostics, Abbott Park, IL, USA)

† The total number of observations was 15,655 in Northern Sweden, 8002 in FINRISK 1997, 14,902 in SHHEC, 8393 in MONICA/KORA, 4782 in MONICA Brianza, 24,266 in Moli-sani, 3524 in MATISS, and 5242 in MONICA-Catalonia

‡ Reasons for missing data:

Northern Sweden—technical issues (71.3%), too small sample volume (23.5%), and non-available sample (5.2%)

FINRISK—technical issues (41.9%), non-available sample (28.8%), too small sample volume (22.8%), and sample mix-up (6.5%)

SHHEC—too small sample volume (76.8%), non-available sample (20.0%), technical issues (1.8%), and non-available reagent (1.4%)

MONICA/KORA—too small sample volume (74.1%), technical issues (14.6%), unspecified sample issues (7.0%), non-available sample (3.8%), and sample mix-up (0.5%)

MONICA Brianza—too small sample volume (69.3%), unspecified sample issues (24.6%), non-available sample (3.1%), technical issues (2.7%), and sample mix-up (0.3%)

Moli-sani—too small sample volume (31.6%), technical issues (26.4%), non-available sample (25.6%), unspecified sample issues (13.2%), and sample mix-up (3.2%)

MATISS—too small sample volume (80.3%), technical issues (17.1%), and non-available sample (2.6%)

MONICA-Catalonia—too small sample volume (60.8%), non-available sample (28.3%), technical issues (8.0%), and unspecified sample issues (2.9%)

Online Resource 5 Weekly variation of 25-hydroxyvitamin D (25[OH]D) status in the study population with complete data on 25(OH)D (*n* = 77,320, including 80,934 observations). The *upper panel* shows the observed median concentration for each calendar week of sampling and the *lower panel* shows the quantile regression-derived difference in median concentration by calendar week of sampling (modeled as a continuous variable by using 4-knot restricted cubic splines [knots at the 5th, 35th, 65th, and 95th percentile] and with the comparator value set to calendar week 12)

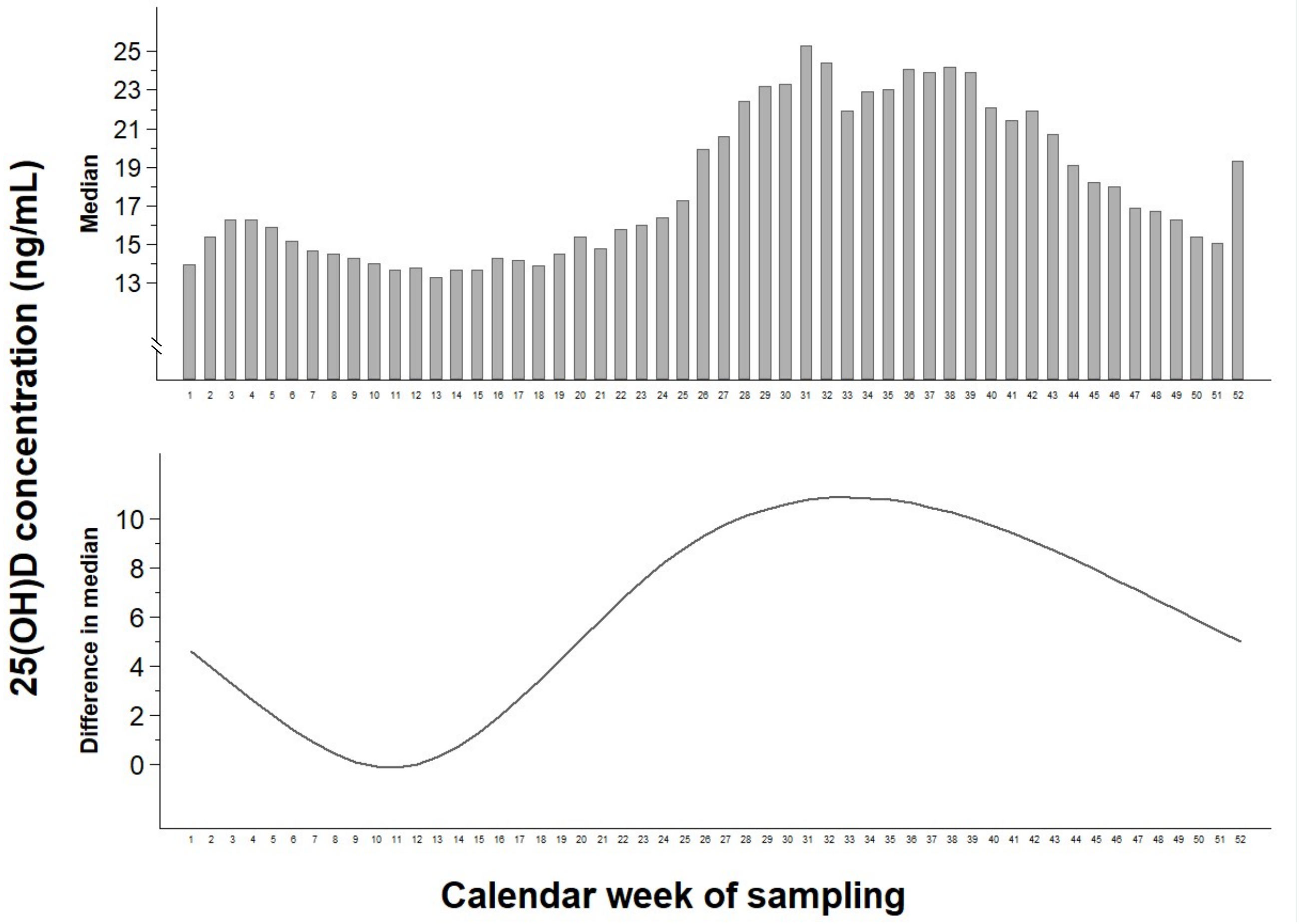

Online Resource 6 Baseline characteristics of the Italian study population (n = 32,572) by cohort

	Cohort		
Characteristics	MONICA Brianza	MATISS	Moli-sani
No. of participants	4782	3524	24,266
Participant characteristics			
Median age (range) (years)	46.6 (25.6, 66.6)	50.4 (20.6, 81.4)	54.6 (34.6, 98.7)
Male sex (%)	49.3	34.3	48.1
Decade of sampling (%)			
1980s	57.8	–	–
1990s	42.2	100	–
2000s	–	–	100
Season of sampling (%)			
Winter (December to February)	29.3	19.9	22.1
Spring (March to May)	17.9	27.4	31.0
Summer (June to August)	19.0	13.3	20.9
Fall (September to November)	33.8	39.4	26.0
Blood sample characteristics			
Median time to analysis (range) (years)	25.3 (20.2, 28.8)	21.5 (19.9, 22.8)	7.1 (4.5, 9.4)
Type of sampling material (%)			
Serum	100	100	100
Plasma	–	–	–

MONICA, Monitoring of Trends and Determinants in Cardiovascular disease; MATISS, Malattie Aterosclerotiche Istituto Superiore di Sanità

Online Resource 7 Observed variation of 25-hydroxyvitamin D (25[OH]D) status in the study population with complete data on 25(OH)D (n = 77,320, including 80,934 observations) by decade (1980s, 1990s, and 2000s) and season of sampling (winter, spring, summer, and fall). The included cohorts contributed with data to each subgroup as follows: Sweden (1980s, 1990s, and 2000s; winter and spring), Finland (1990s; winter and spring), Scotland (1980s and 1990s; winter, spring, summer, and fall), Germany (1990s and 2000s; winter, spring, summer, and fall), Italy (1980s, 1990s, and 2000s; winter, spring, summer, and fall), and Spain (1980s and 1990s; winter, spring, summer, and fall)

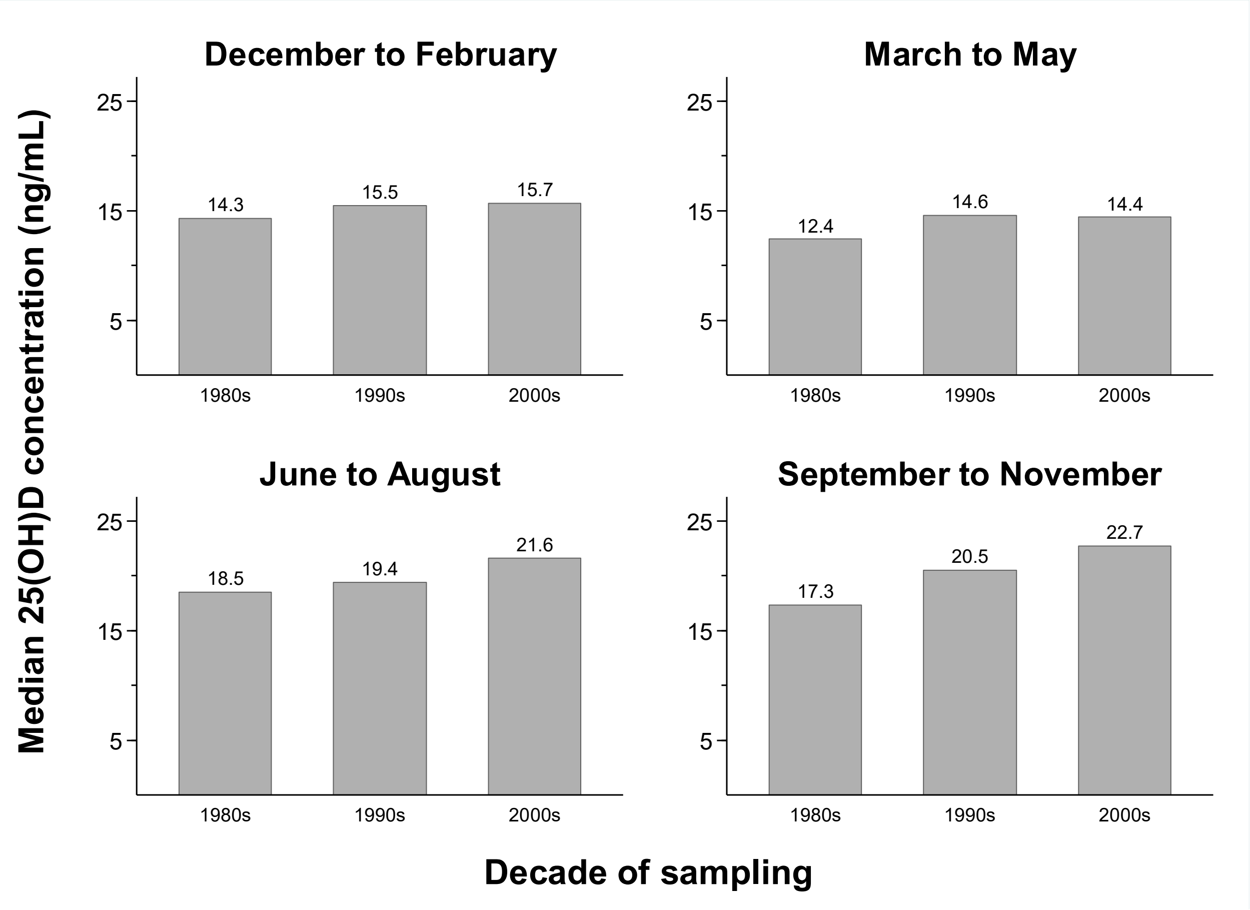

Online Resource 8 Differences in 25(OH)D status in the study population by country and based on complete data on 25(OH)D (*n* = 77,320, including 80,934 observations)

	Country (ordered by decreasing geographical latitude)					
25(OH)D status (ng/mL)	Sweden	Finland	Scotland	Germany	Italy	Spain
Percentile difference*						
5th	Comp. (95% CI)	-2.04 (-2.36, -1.72)	-3.55 (-3.79, -3.30)	-0.93 (-1.21, -0.66)	-3.15 (-3.36, -2.95)	-5.64 (-5.88, -5.40)
10th	Comp. (95% CI)	-2.39 (-2.68, -2.10)	-3.99 (-4.23, -3.75)	-1.19 (-1.44, -0.94)	-3.64 (-3.82, -3.45)	-6.34 (-6.59, -6.09)
25th	Comp. (95% CI)	-2.93 (-3.22, -2.63)	-5.29 (-5.53, -5.05)	-2.35 (-2.59, -2.10)	-4.61 (-4.79, -4.43)	-7.72 (-7.96, -7.49)
50th	Comp. (95% CI)	-3.28 (-3.64, -2.92)	-6.94 (-7.24, -6.65)	-3.15 (-3.44, -2.86)	-5.51 (-5.71, -5.30)	-9.34 (-9.63, -9.05)
75th	Comp. (95% CI)	-3.57 (-4.05, -3.09)	-8.36 (-8.75, -7.97)	-4.03 (-4.41, -3.65)	-6.39 (-6.66, -6.13)	-10.81 (-11.21, -10.41)
90th	Comp. (95% CI)	-3.75 (-4.48, -3.02)	-9.48 (-10.11, -8.85)	-4.55 (-5.18, -3.91)	-7.61 (-8.05, -7.17)	-12.43 (-13.02, -11.84)
95th	Comp. (95% CI)	-4.46 (-5.52, -3.40)	-10.09 (-11.00, -9.18)	-5.00 (-5.89, -4.11)	-8.67 (-9.32, -8.01)	-13.49 (-14.42, -12.57)

25(OH)D, 25-hydroxyvitamin D; Comp., comparator; CI, confidence interval

* Estimated from quantile regression models adjusted for sex, age (continuous using 4-knot restricted cubic splines [4-RCS], years), calendar week of sampling (continuous using 4-RCS, calendar weeks), decade of sampling (1980s, 1990s, 2000s), and time from sampling to analysis (continuous using 4-RCS, years)
